# Genomic alteration of MTAP/CDKN2A predicts sarcomatoid differentiation and poor prognosis and modulates response to immune checkpoint blockade in renal cell carcinoma

**DOI:** 10.3389/fimmu.2022.953721

**Published:** 2022-08-01

**Authors:** Wenhao Xu, Aihetaimujiang Anwaier, Wangrui Liu, Gaomeng Wei, Jiaqi Su, Xi Tian, Jing Xia, Yuanyuan Qu, Jianyuan Zhao, Hailiang Zhang, Dingwei Ye

**Affiliations:** ^1^ Department of Urology, Fudan University Shanghai Cancer Center, Shanghai, China; ^2^ Department of Interventional Oncology, Renji Hospital, Shanghai Jiao Tong University School of Medicine, Shanghai, China; ^3^ Affiliated Hospital of Youjiang Medical University for Nationalities, Baise, China; ^4^ The Medical Department, 3D Medicines Inc., Shanghai, China; ^5^ Institute for Developmental and Regenerative Cardiovascular Medicine, MOE-Shanghai Key Laboratory of Children’s Environmental Health, Xinhua Hospital, Shanghai Jiao Tong University School of Medicine, Shanghai, China

**Keywords:** renal cell carcinoma, sarcomatoid differentiation, immunotherapy, genomic alteration, MTAP, CDKN2A, tumor microenvironment

## Abstract

Sarcomatoid differentiation is a highly aggressive pathological characteristic of renal cell carcinoma (RCC) and is characterized by susceptibility to progression and extremely poor prognosis. In this study, we included all genomic alteration events that led to a loss of protein function of MTAP and CDKN2A, and enrolled 5,307 RCC patients with genomic sequencing data from Western and Chinese cohorts. Notably, *MTAP*/*CDKN2A* ^MUT^ occurred in the Chinese population ~2 times more frequently than in the Western cohort and showed significant co-mutation trends. We found significantly higher proportions of sarcomatoid-positive patients with *MTAP* ^MUT^ or *CDKN2A* ^MUT^ compared with *MTAP*/*CDKN2A* wild-type (WT) patients (*P* < 0.001). Of the 574 RCC samples from the FUSCC cohort and 3,563 RCC samples from 17 independent cohorts, the *MTAP*/*CDKN2A* ^MUT^ significantly predicted extremely poor outcomes (*P* < 0.0001). The Western cohort suggested a concordant relationship between *MTAP*/*CDKN2A* ^MUT^ and sarcomatoid differentiation in RCC. Moreover, although *MTAP*/*CDKN2A* ^MUT^ RCC may be insensitive to targeted therapy, the high degree of tumor heterogeneity and higher PD-L1 and CXCL13 expression characterizations reflected that *MTAP*/*CDKN2A*-deficient features could benefit from immunotherapy for patients with RCC. This study utilized RCC samples from large-scale, global, multicenter sequencing cohorts and first proved that *MTAP*/*CDKN2A* deficiency significantly correlates with sarcomatoid differentiation in RCC and predicts aggressive progression, poor prognosis, and primary resistance to targeted therapy and potential favorable responses to immune checkpoint blockade. Unlike conventional targeted therapies, emerging drugs such as immunotherapies or synthetic lethal PRMT5 inhibitors may become novel therapeutic options for patients with *MTAP/CDKN2A* ^MUT^ RCC.

## Introduction

Renal cell carcinoma (RCC) is one of the most common genitourinary malignancies (1), accounting for 3% of all malignant tumors, and its incidence is increasing at a rate of 3% every year (2). The incidence rate in major domestic cities of China, such as Beijing, Shanghai, and Hangzhou, has reached more than 8/100,000, which is more than double that of 10 years ago (3, 4). Clear cell renal cell carcinoma (ccRCC) is the predominant pathological type of kidney cancer, which accounts for about 78% of all RCC in adults. Nearly one-third of ccRCC patients encountered lymphatic, bone, or organ metastases at initial diagnosis, and the 5-year survival rate of patients with advanced ccRCC is less than 20% (5, 6). Although classic histological heterogeneity has been widely explored in the research of ccRCC, the latest advances in genomic technologies have demonstrated prominent molecular subtypes, which have assisted in elucidating the precise typing and treatment of ccRCC as well as mechanisms underlying the inevitable occurrence and development essence (7, 8). Therefore, the multi-omics approach from molecular and genomic levels have become important research techniques in the systematic study of tumor occurrence and treatment efficacy improvement for patients with ccRCC.

Sarcomatoid differentiation is a highly aggressive pathological and extremely uncommon characteristic of RCC and is characterized by susceptibility to metastasis and recurrence and extremely poor prognosis. Renal cell carcinoma with sarcomatoid dedifferentiation (sRCC) is insensitive to chemoradiotherapy and targeted therapy, and radical resection is the preferred treatment. Patients who have clear cell histology and a higher percentage of sarcomatoid differentiation may have worse outcomes with VEGF-targeted therapy (9). In addition, the more sarcoma components, the worse the prognosis of the patient. Even with active treatment, the median postoperative survival for patients with sRCC was still not optimistic (10). For example, with over 42 months of median follow-up, RCC patients with sarcomatoid histology who received nivolumab plus ipilimumab still had median overall survival that was not yet reached [(25.2–not estimable); *n* = 74] versus those who received sunitinib [14.2 months (9.3–22.9); *n* = 65; *P* = 0.0004] (11). Meanwhile, the JAVELIN Renal 101 trial enrolled 108 RCC patients with sarcomatoid histology (47 patients in the avelumab plus axitinib arm and 61 in the sunitinib arm), and patients with sRCC in the combination arm had improved efficacy outcomes versus those in the sunitinib arm (12). The median progression-free survival (PFS) was 7.0 months (95% CI, 5.3–13.8 months) versus 4.0 months (95% CI, 2.7–5.7 months), respectively. Although researchers keep on exploring the unique molecular pathogenesis and driver mutation spectrum of sRCC, the need for individual diagnostic or effective treatment options is urgent (13).

Methylthioadenosine (MTA) phosphorylase (MTAP) is a key enzyme of the methionine remediation synthesis pathway. In MTAP-deficient cancers, MTA accumulation selectively inhibits protein arginine methyltransferase 5 (PRMT5) enzyme activity and increases PRMT5 inhibition sensitivity (14). Precisely because of this synthetic lethal mechanism, the development and application of drugs targeting PRMT5 have led to new treatment options for patients with MTAP deletion tumors (15). Since *MTAP* is located on chromosome 9p21, close to the tumor suppressor *CDKN2A*, genomic alteration of *MTAP*/*CDKN2A* co-occurrence is frequent. At the EAU2021 conference, Necchi et al. compared genomic differences between sRCC and clear cell RCC (ccRCC). The authors found a significant increase in *MTAP*/*CDKN2A* loss in sRCC and proposed a potential role of anti-PRMT5 drugs in MTAP-deficient advanced sRCC (16). However, the lack of clinical data and the small sample size were major limitations.

In this study, we included all genomic alteration (GA) events that led to a loss of protein function in the statistical analysis. The study included 574 Chinese patients with RCC from the Fudan University Shanghai Cancer Center (FUSCC); 3,563 RCC samples from 17 independent cohorts were integrated (Western). *MTAP*/*CDKN2A* alteration frequency, clinicopathological features, and prognosis were depicted in the FUSCC and Western cohorts. Finally, we included 1,170 Chinese RCC patients from the 3D cohort with panel sequencing data. The relationship of *CDKN2A* mutations with therapeutic markers was further explored. We hypothesized that *MTAP*/*CDKN2A*-deficient features significantly correlate with sarcomatoid differentiation in RCC and predict poor outcomes, inactive targeted treatment responsiveness, and favorable responses to immune checkpoint blockade in patients with RCC.

## Methods

### Data collection and preprocessing from the discovery, testing, and validation sets

In the Chinese training cohort, 574 patients with available whole-exome sequencing (WES) data from the FUSCC (Shanghai, China) were included. In the Western testing cohort, the WES sequencing data of 3,563 ccRCC Caucasian patients were obtained from the cBioPortal for Cancer Genomics database (http://www.cbioportal.org/) with gene IDs converted from Ensembl ID to gene symbol matrix. The combined 3D medicine cohort included WES data of DNA extracted from clinically annotated tumor specimens and from whole blood (as the matched germline source) from a total of 1,170 Chinese RCC patients.

### Genetic variation analysis

The somatic mutation data were processed using the “maftools” R package to screen and present the genes with the top 20 mutation frequencies. The chi-square test was implemented to analyze the differences in the mutation frequencies of the high-frequency mutant genes among the three cohorts. We counted all genomic alterations (specifically the focal loss of 9p21) to the *MTAP* and *CDKN2A* genes that sit next to each other on chromosome 9p21 and referred to the altered status as “MUT.”

### Clinicopathological subgroup analysis

We divided patients into different subgroups based on different clinicopathological features, including neoplasm histological grade, cancer metastasis, cancer lymph node stage, neoplasm clinical stage, and race category; different hemogram features, including the levels of hemoglobin, platelet, WBC, and serum calcium; and different clinical therapy data, including individual neoplasm status, sunitinib treatment after operation, new neoplasm event after initial therapy indicator, and response to systematic first-line targeted therapy. The Fisher test was used to compare the differences in different clinical subgroups between the *MTAP*/*CDKN2A*-altered and *MTAP*/*CDKN2A*-unaltered groups.

### Differential gene expression analysis and functional enrichment analysis

To explore the potential biological differences between the *MTAP*/*CDKN2A*-altered and *MTAP*/*CDKN2A*-unaltered patterns, the “limma” R package was used to identify differentially expressed genes (DEGs), and the threshold value was set as *P <*0.05, |logFC| ≥3.37. Functional enrichment analyses were carried out to explore the potential functions of the genomic alteration of *MTAP*/*CDKN2A* in patients with RCC using the Gene Ontology (GO), Kyoto Encyclopedia of Genes and Genomes (KEGG), and Reactome databases (17).

### Hematoxylin and eosin and immunohistochemistry staining analysis

Hematoxylin and eosin staining was conducted according to routine protocols. Briefly, after deparaffinization and rehydration, tissue sections were stained with hematoxylin solution and eosin solution (ZSGB-BIO, China), followed by dehydration with graded alcohol and clearing in xylene. The mounted slides were then examined and photographed using a LEICA DM3000 LED (Leica DMshare (v3), Germany) following the manufacturer’s protocols and previously described procedure (8). IHC was performed to evaluate the expression level of CXCL13 (1:1,000 dilution; ab246518; Abcam, USA) and PD-L1 (1:300 dilution; No. 19313684, CST, China) in ccRCC samples from the FUSCC according to standard procedures as previously described (8). Staining score and sarcomatoid differentiation features were independently measured by two experienced clinical pathologists.

### Statistical analysis

In the statistical analyses, the Wilcoxon test was used to compare the differences between the two groups of samples. The survival curve was analyzed using Kaplan–Meier, and the log-rank test was used to assess the significance for disease-specific survival (DSS), disease-free survival (DFS), overall survival (OS), and PFS. The “survminer” R package was utilized to take the best cutoff value for all survival analyses. A *P*-value less than 0.05 was considered statistically significant.

## Results

### Clinical value of MTAP/CDKN2A^MUT^ in sarcomatoid and prognosis of 574 patients with RCC from the FUSCC cohort

**Figure 1:**
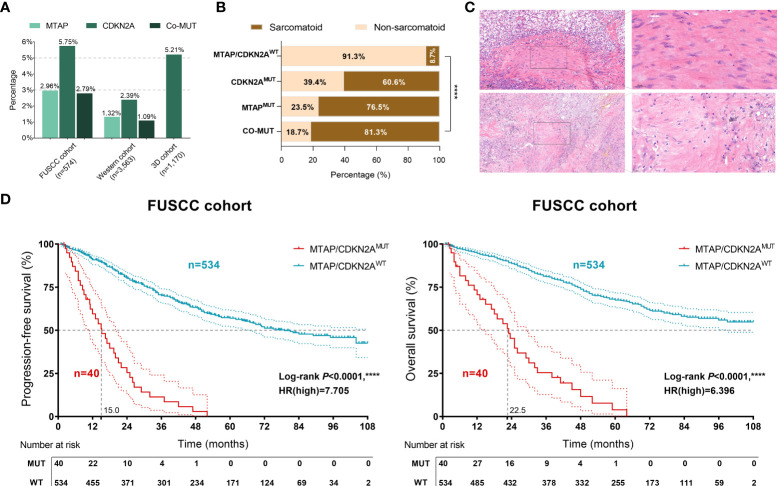
Clinical value of *MTAP*/*CDKN2A* ^MUT^ in sarcomatoid and the prognosis of 574 patients with renal cell carcinoma (RCC) from the discovery set of the Fudan University Shanghai Cancer Center (FUSCC) cohort. **(A)** Genomic alteration frequency of *MTAP* and *CDKN2A* in 574 Chinese patients with RCC from the FUSCC cohort; 3,563 RCC samples from 17 independent cohorts were integrated (Western cohort), and 1,170 Chinese patients with RCC were included from the 3D medicine cohort with panel sequencing data. **(B)** The proportion of sarcomatoid pathological features in 574 patients with RCC from the FUSCC cohort in different mutation groups. **(C)** The representative images of sarcomatoid pathological features in *MTAP*/*CDKN2A^MUT^* samples. **(D)** Classified by the *MTAP*/*CDKN2A^MUT^*, progression-free survival (PFS) and overall survival (OS) of 574 RCC patients from the FUSCC cohort using Kaplan–Meier curve analysis. ****, P<0.0001.

First, we summarized the mutation frequencies of *MTAP*/*CDKN2A* in the three cohorts, namely, the discovery set (FUSCC cohort, *n* = 574), the testing set (Western cohort, *n* = 3,563), and the validation cohort (3D medicine cohort, *n* = 1,170) (**Figure 1A**). The results showed the highest frequency of *MTAP* ^MUT^ and *CDKN2A* ^MUT^ (2.96% and 5.75%, respectively) in the FUSCC cohort, and the *MTAP* ^MUT^ frequency was approximately half of *CDKN2A* ^MUT^ frequency. Notably, *MTAP*/*CDKN2A* ^MUT^ occurred in the Chinese population ~2 times more frequently than in the Western cohort and showed significant co-mutation trends.

To verify the relationship between *MTAP*/*CDKN2A* mutations and sarcomatoid differentiation, we evaluated the pathological features of 574 samples from the FUSCC cohort and found significantly higher proportions of sarcomatoid-positive patients with *MTAP* ^MUT^ or *CDKN2A* ^MUT^ compared with *MTAP*/*CDKN2A* wild-type (WT) patients (*P* < 0.001; **Figure 1B**). The majority of *MTAP*/*CDKN2A* ^MUT^ samples showed sarcomatoid pathological features (**Figure 1C**). Of the 574 RCC samples, the *MTAP*/*CDKN2A* ^MUT^ significantly predicted extremely poor PFS [hazard ratio (HR)=7.705, *P* < 0.0001] and OS (HR = 6.369, *P* < 0.0001; **Figure 1D**). Taken together, we found that *MTAP*/*CDKN2A* ^MUT^ could significantly predict sarcomatoid differentiation and prognosis in RCC.

### MTAP/CDKN2A^MUT^ in histopathological subtypes and sarcomatoid differentiation of 3,563 patients with RCC from the Western cohort

**Figure 2:**
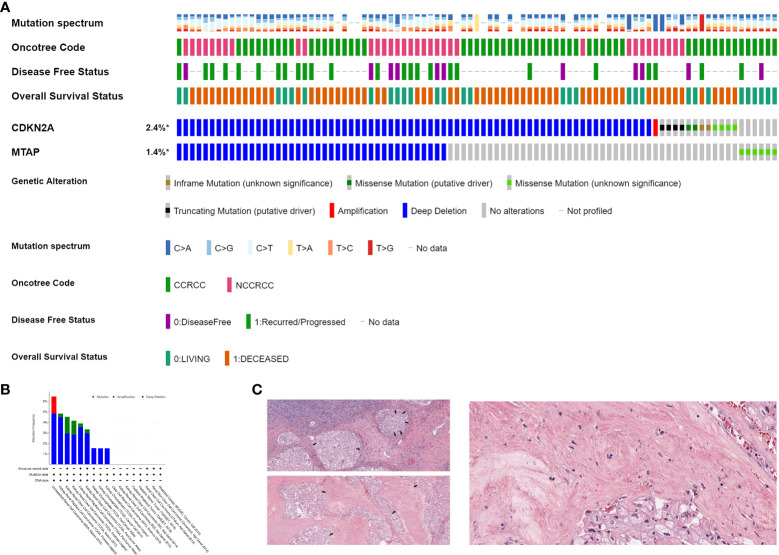
*MTAP*/*CDKN2A* ^MUT^ in histopathological subtypes and sarcomatoid differentiation of 3,563 patients with RCC from the Western cohort. **(A)** Relationship between *MTAP*/*CDKN2A* ^MUT^ and histopathological subtypes of RCC, disease-free status, and OS status in 3,563 RCC samples from the Western cohort. **(B)** The frequency of *MTAP*/*CDKN2A* genomic alterations in different RCC datasets. **(C)** Remarkable sarcomatoid differentiation in patients with both *MTAP* ^MUT^ and *CDKN2A* ^MUT^ from The Cancer Genome Atlas. * Ratio is calculated as altered/profiled.

To further test our hypothesis, we then explored *MTAP*/*CDKN2A* ^MUT^ frequencies in 3,563 RCC samples from the Western cohort. As shown in **Figure 2A**, *MTAP*/*CDKN2A* ^MUT^ is more common in non-ccRCC, and the frequency of progression and mortality events was significantly increased in the GA group. The frequency of *MTAP*/*CDKN2A* ^MUT^ in different RCC datasets is shown in **Figure 2B**. The results revealed that *MTAP*/*CDKN2A* ^MUT^ is more common in non-ccRCC histopathological subtypes, which is consistent with the consensus that sarcomatoid differentiation occurs more frequently in non-ccRCC. Interestingly, we found remarkable sarcomatoid differentiation in patients with both *MTAP* ^MUT^ and *CDKN2A* ^MUT^ (**Figure 2C**). Overall, the results from the Western cohort suggest a concordant relationship between *MTAP*/*CDKN2A* ^MUT^ and sarcomatoid differentiation in RCC.

### Implications of MTAP/CDKN2A^MUT^ in prognosis, clinicopathological features, and sensitivity to therapy of 3,563 patients with RCC from the Western cohort

**Figure 3:**
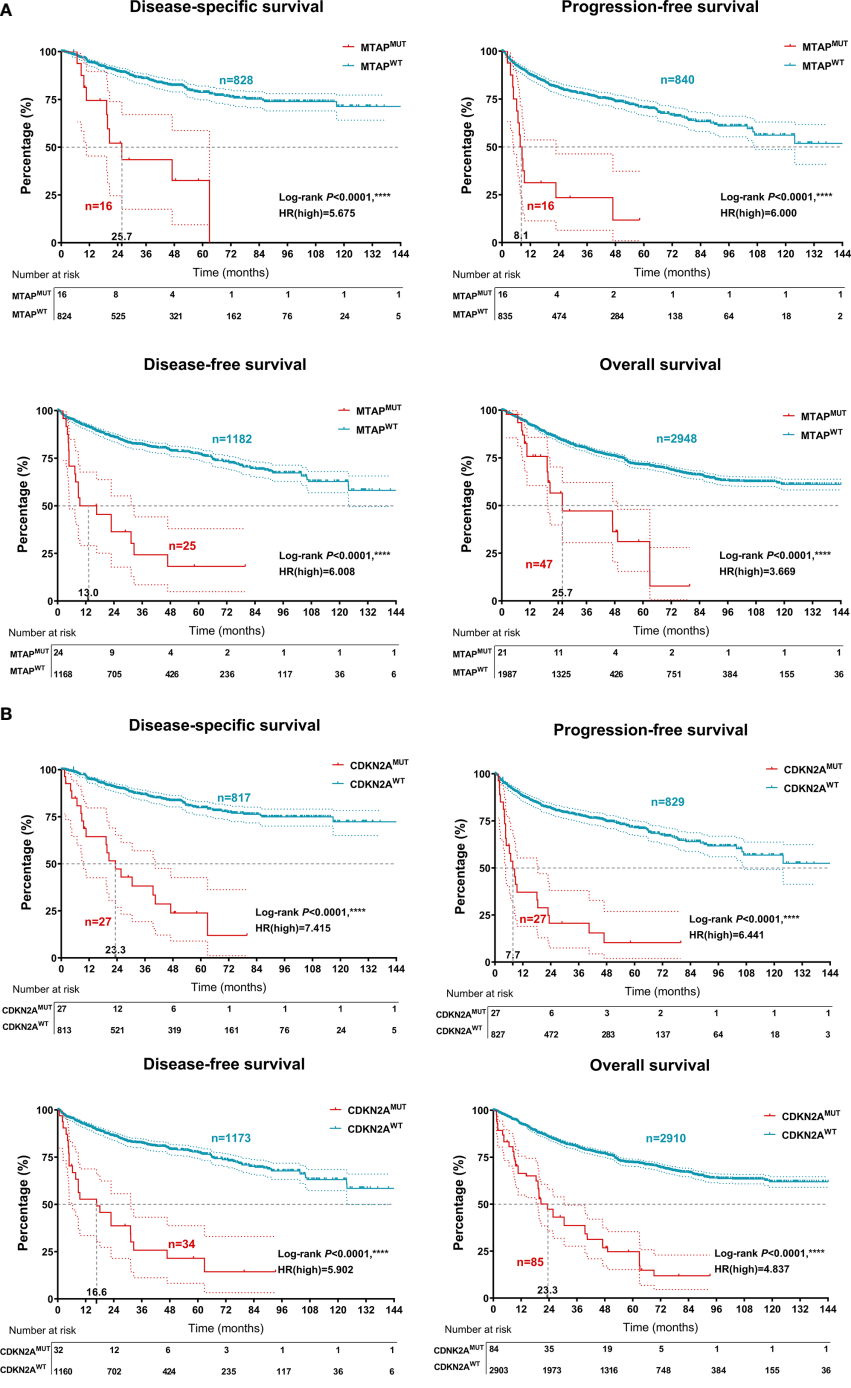
Prognostic implications of *MTAP* ^MUT^ and *CDKN2A* ^MUT^ 3,563 patients with RCC from the testing set of the Western cohort. **(A)** Classified by the *MTAP*/*CDKN2A^MUT^*, disease-specific survival, PFS, disease-free survival, and OS of RCC patients from the Western cohort using Kaplan–Meier survival analysis. **(B)** Association between *MTAP*/*CDKN2A^MUT^* and clinicopathological characteristics, association between *MTAP*/*CDKN2A^MUT^* and hemogram, and association between *MTAP*/*CDKN2A^MUT^* and clinical therapeutic efficacy. ****, P<0.0001.

Next, we collected RCC samples with available survival and mutation information from the Western cohort. Significantly, the genomic alteration of *MTAP* prominently predicted shorter DSS (*P* < 0.0001, HR=5.675), PFS (*P* < 0.0001, HR = 6.000), DFS (*P* < 0.0001, HR = 6.008), and OS (*P* < 0.0001, HR = 3.669) in patients with RCC from the Western testing cohort (**Figure 3A**). Moreover, the genomic alteration of *CDKN2A* was also significantly associated with shorter DSS (*P* < 0.0001, HR = 7.415), PFS (*P* < 0.0001, HR = 6.441), DFS (*P* < 0.0001, HR = 5.902), and OS (*P* < 0.0001, HR = 4.837) in 3,563 patients with RCC from the Western cohort (**Figure 3B**).

**Figure 4:**
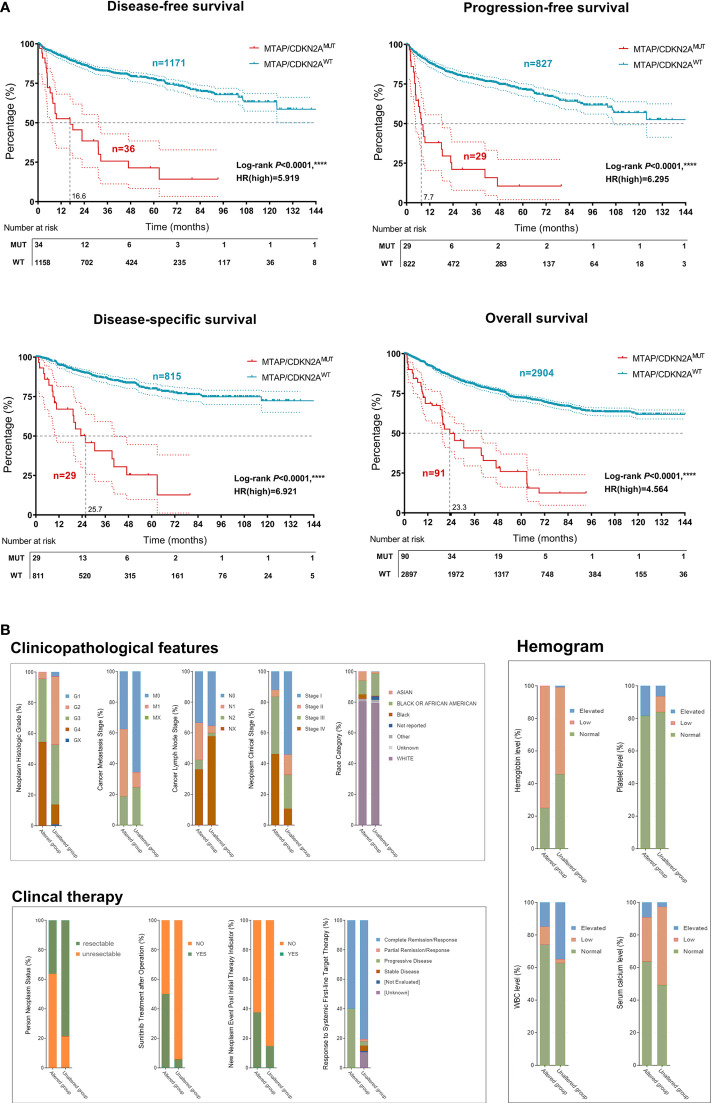
Implications of *MTAP*/*CDKN2A* ^MUT^ in prognosis, clinicopathological features, and sensitivity to therapy of 3,563 patients with RCC from the Western cohort. **(A)** Classified by the *MTAP^MUT^*, DSS, PFS, DFS, and OS of RCC patients from the Western cohort using Kaplan–Meier survival analysis and log-rank test. **(B)** Classified by the *CDKN2A^MUT^*, DSS, PFS, DFS, and OS of RCC patients from the Western cohort using Kaplan–Meier survival analysis and log-rank test. ****, P<0.0001.

Additionally, the results revealed that *MTAP*/*CDKN2A* ^MUT^ significantly predicted poor DSS (*P* < 0.0001, HR = 6.921), PFS (*P* < 0.0001, HR = 6.295), DFS (*P* < 0.0001, HR = 5.919), and OS (*P* < 0.0001, HR = 4.564) in patients with RCC from the testing set (**Figure 4A**). Moreover, RCC patients with *MTAP*/*CDKN2A* ^MUT^ showed significantly higher pathology grades (95.8% were G3–G4) and clinical stages (92.5% were III–IV; **Figure 4B**). Consistently, we found a significantly higher frequency of Asian subjects in the GA group than in the unaltered group. Additionally, patients in the GA group exhibited lower hemoglobin and leukocyte levels and higher platelet and blood calcium levels, reflecting a higher IMDC stage and a higher treatment primary tolerance. Additionally, in the GA group, the proportion of patients with unresectable tumors was 63.5%; 50% of the patients received postoperative sunitinib therapy, and approximately 40% of patients carried new neoplasm events after initial therapy. These findings suggested that patients in the GA group are primarily resistant to conventional targeted therapy (**Figures S1A, B**). Overall, to our knowledge, this is the first verification of the prominent relationship between *MTAP*/*CDKN2A* ^MUT^ and sRCC. *MTAP*/*CDKN2A* ^MUT^ could significantly predict progression, long-term survival, and treatment efficacy in RCC patients.

### MTAP/CDKN2A^MUT^ predicts higher tumor heterogeneity, tumor microenvironment characterizations, and active responses to immune checkpoint blockade of RCC patients

**Figure 5:**
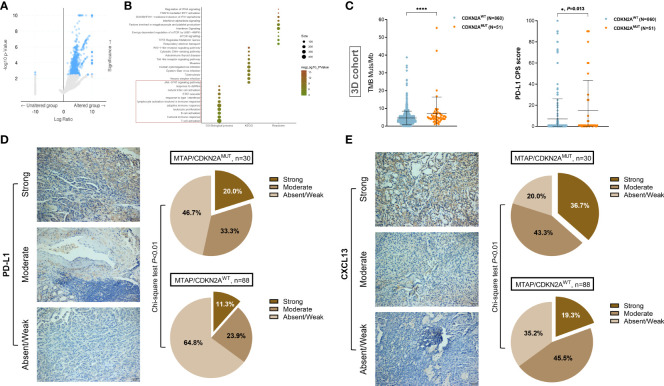
*MTAP*/*CDKN2A* ^MUT^ predicts tumor heterogeneity and TME characterizations of RCC patients from the 3D and FUSCC cohorts. **(A)** Identification of differential altered genes between *MTAP*/*CDKN2A^MUT^* and unaltered groups in the Western cohort using the “limma” R package with a threshold of Log^10^(*q*-value) >2. **(B)** Gene Ontology (GO), Kyoto Encyclopedia of Genes and Genomes (KEGG), and Reactome functional enrichment analyses were performed to speculate on the biological functions and tumor environment changes involved in *MTAP/CDKN2A* ^MUT^. **(C)** Tumor mutation burden (TMB) and PD-L1 levels in the *CDKN2A^MUT^* group compared with the *CDKN2A^WT^* group in 1,170 Chinese patients with RCC from the 3D medicine cohort. **(D, E)** Protein expression levels of immune checkpoint molecule, PD-L1, and the key lymphokine that recruits tertiary lymphoid structures, CXCL13, were evaluated in ccRCC samples with available genomic data from the discovery set of the FUSCC cohort. The relative expression of PD-L1 and CXCL13 was defined as strong, moderate, and absent/weak staining according to the staining density and intensity of each section. The chi-square test was used to compare differences between groups. *, P<0.05; ****, P<0.0001.

The above findings aroused our interest regarding the impact of *MTAP*/*CDKN2A^MUT^* on RCC malignant biological functions. Therefore, we analyzed genotype differences in the Western cohort using the “limma” R package with a threshold of Log^10^(*q*-value) >2. A total of 363 significantly upregulated genes in the *MTAP*/*CDKN2A* ^MUT^ group were identified (**Figure 5A**). Functional enrichment suggested that *MTAP/CDKN2A* ^MUT^ response genes participate in the aggregation and activation of immune cells in the tumor microenvironment (TME). Similarly, pathway enrichment results revealed a marked involvement in the JAK–STAT, interferon, and mTOR signaling pathways, suggesting that *MTAP*/*CDKN2A* ^MUT^ may activate the anti-tumor immune response in the TME of RCC (**Figure 5B**). Notably, the 3D cohort showed significantly elevated tumor mutation burden (TMB) and PD-L1 levels in the *CDKN2A* ^MUT^ group (**Figure 5C**).

To further portray intratumoral immunophenotypes according to genomic alterations of MTAP and CDKN2A, we evaluated the expression levels of the immune checkpoint molecule, PD-L1, and the key lymphokine that recruits tertiary lymphoid structures, CXCL13, in ccRCC samples with available genomic data from the discovery set of the FUSCC cohort. The relative expression of PD-L1 and CXCL13 was defined as strong, moderate, and absent/weak staining according to the staining density and intensity of each section. Interestingly, significantly higher PD-L1 and CXCL13 expression was found in 30 RCC samples with *MTAP*/*CDKN2A* ^MUT^ compared with 88 patients in the *MTAP*/*CDKN2A* ^WT^ group (*P* < 0.01; **Figures 5D, E**). In general, these results demonstrated that although *MTAP*/*CDKN2A* ^MUT^ RCC may be insensitive to targeted therapy, the high degree of tumor heterogeneity and immune-excluded TME reflected that *MTAP*/*CDKN2A^MUT^* patients could benefit from immunotherapies.

## Discussion

The advancement of cancer genome analyses has revealed the detailed genomic landscapes of cancer. Targeted therapies based on the characteristics of the tumor genome are increasingly being offered to patients with cancers (18–20). Our study has essential implications for the design and analysis of molecular epidemiology studies in patients with sarcomatoid differentiation of RCC as well as the somatic characterization of genomes for the Chinese population. Among the Chinese and Caucasian populations, we assessed the mutation landscape and found the highest frequency of *MTAP* ^MUT^ and *CDKN2A* ^MUT^ (2.96% and 5.75%, respectively) in the FUSCC cohort, and the *MTAP* ^MUT^ frequency was approximately half of *CDKN2A* ^MUT^ frequency. Notably, *MTAP/CDKN2A* ^MUT^ occurred in the Chinese population ~2 times more frequently than in the Western cohort and showed significant co-mutation trends.

A previous study identified *VHL* mutations as the most common gene mutation (72%), followed by *PBRM1* (45%), *SETD2* (34%), and *BAP1* mutations (17%) in 29 Caucasian patients with advanced ccRCC (21). It is also suggested that *BAP1*, *SETD2*, and *PBRM1* are prevalent co-drivers of tumor grade and invasion of ccRCC and associated with aggressive progression (21–23). However, PBRM1-mutant patients tended to have a higher TMB and might evoke immunotherapy sensitivity (24). Furthermore, Malouf et al. detected genomic profiling, underpinning renal cell carcinoma with sarcomatoid dedifferentiation, and identified *TP53* (42.3%), *VHL* (34.6%), *CDKN2A* (26.9%), and *NF2* (19.2%) as the most frequently altered genes for sRCC (12, 25). Approaches and strategies are needed for other malignancies driven by the loss of CDKN2 and NF2, as revealed by genomic features in the course of clinical management for patients with sRCC. This study speculated that although the initiating mutation in sRCC is similar to other types of RCC, the acquisition of other driver alterations, such as TP53 or NF2, may lead to the generation of the sarcomatoid phenotype. Therefore, understanding the distinct genomic alteration background of the Chinese population with sarcomatoid differentiation of RCC will guide targeted therapies and immunotherapies, paving the way for the clinical practice of precision medicine in this highly lethal cancer.

Previous studies have revealed the unique molecular classification of ccRCC predicting prognosis in Caucasian patients treated with targeted therapies, specifically the TKIs (26). The molecular classification (ccRCC1–4) suggested a high predictive value for favorable outcomes in patients with advanced ccRCC treated with sunitinib (27). This study identified the clusters with *MTAP*/*CDKN2A*-altered and *MTAP*/*CDKN2A*-unaltered groups with distinct levels of genomic alteration, clinicopathological features, and immune infiltration in patients with RCC. Moreover, RCC patients with *MTAP*/*CDKN2A* ^MUT^ showed significantly higher pathology grades (95.8% were G3–G4) and clinical stages (92.5% were III–IV). Consistently, we found a significantly higher frequency of Asian subjects in the GA group than in the unaltered group. Additionally, patients in the GA group exhibited lower hemoglobin and leukocyte levels and higher platelet and blood calcium levels, reflecting a higher IMDC stage and a higher treatment primary tolerance. Additionally, in the GA group, the proportion of patients with unresectable tumors was 63.5%; 50% of the patients received postoperative sunitinib therapy, and approximately 40% of patients carried new neoplasm events after initial therapy.

The loss of MTAP expression has been linked to a tumor-promoting effect in multiple cancers, and MTAP may serve as a tumor suppressor (28–30). For example, in 2021, Han et al. found that 9p21 loss, normally CDKN2A (13.5%) and MTAP (9.3%), confers a cold tumor immune microenvironment, poor clinical outcomes, and primary resistance to immune checkpoint therapy for cancers (31). However, this study did not explore the clinicopathological significance of 9p21 deletion in RCC, let alone its impact on the dysregulation of TME. Despite the frequent loss of MTAP expression in high-grade gliomas, MTAP did not appear to be associated with a deteriorating outcome, and *in-vitro* models demonstrated that MTAP did not affect proliferation, invasion, and migration (32). Unlike in other tumors, non-synonymous mutations, neoantigens, insertions, or deletions caused by chromosomal structural changes and somatic copy number variations were not in significant consistency with the response to immune checkpoint therapies (ICTs) in ccRCC (33). Moreover, the elevated expression of PD-L1 correlated with poor OS, which was also observed in the pro-tumorigenic *MTAP/CDKN2A* ^MUT^ cluster (34, 35).

A previous study pointed out that genetic alteration of CDKN2A was correlated with reduced benefits from ICTs and changes in the TME of urothelial carcinoma (36). Furthermore, CDKN2A deletion and BAP1 mutations, as well as increased expression of MYC transcriptional programs, have been identified as genomic features of aggressive behavior for sarcomatoid and rhabdoid RCC (37). In this study, the relatively immune-infiltrated *MTAP/CDKN2A* ^MUT^ cluster showed a transcriptional signature indicative of pro-tumorigenic immune infiltration in tumors and prominently higher TMB values based on 1,170 Chinese patients with RCC. Interestingly, we identified the mutually exclusive aggressive tumor phenotypes in ccRCC. Through phenotypic analysis, favorable clinical response to ICTs, elevated expression of immune checkpoints, increased abundance of tumor-infiltrated lymphocyte infiltration, and elevated TMB and PD-L1 expression were observed in the *MTAP/CDKN2A* ^MUT^ cluster. However, just as not all solid tumors with high TMB are sensitive to ICTs, patients with high neoantigens are not necessarily accompanied by an elevated level of CD8^+^ T-cell infiltration in the TME of ccRCC (38). Therefore, under a paradigm of targeted therapies, such as TKIs, two of the clusters that are “immune-infiltrated TME” exerted poorer prognosis but might be uniquely responsive to immune checkpoint blockade, thereby improving treatment outcomes for ccRCC patients. Further tumor type-specific studies are warranted in investigating biomarkers for ICTs.

## Conclusion

This study utilized RCC samples from large-scale, global, multicenter sequencing cohorts and first proved that genomic alteration of *MTAP*/*CDKN2A* significantly correlates with sarcomatoid differentiation in RCC and predicts aggressive progression, poor prognosis, primary resistance to targeted therapy, and potential favorable responses to immune checkpoint blockade. Unlike conventional targeted therapies, emerging drugs such as immunotherapies or synthetic lethal PRMT5 inhibitors may become novel therapeutic options for patients with *MTAP*/*CDKN2A* ^MUT^ RCC.

## Data availability statement

The original contributions presented in the study are included in the article/**Supplementary Material**. Further inquiries can be directed to the corresponding authors.

## Ethics statement

The studies involving human participants were reviewed and approved by ethics committee of Fudan University Shanghai Cancer Center (ID: 050432-4-1805C, Shanghai, China). The patients/participants provided their written informed consent to participate in this study.

## Author contributions

Conceptualization: WX, AA, GW, and WL. Data curation and formal analysis: WX, WL, AA, GW, JX, W Shi, JS, and XT. Funding acquisition: WX, YQ, HZ, and DY. Investigation and methodology: WX, AA, WL, GW, JZ, and HZ. Resources and software: JZ, YQ, HZ, and DY. Supervision: JZ, HZ, and DY. Validation and visualization: WX, AA, WL, and JS. Original draft: WX, AA, WL, and GW. Editing: JZ, HZ, and DY. All authors contributed to the article and approved the submitted version.

## Funding

This work was supported by grants from the “Eagle” Program of Shanghai Anticancer Association (No. SHCY-JC-2021105), the Natural Science Foundation of Shanghai (No. 20ZR1413100). and Shanghai Municipal Health Bureau (No. 2020CXJQ03).

## Acknowledgments

We are grateful to all patients for their dedicated participation in the current study. We expressed our sincere gratitude to Ms. Zoo for editing the figures for this study.

## Conflict of interest

Author JX was employed by The Medical Department, 3D Medicines Inc., Shanghai, China.

The remaining authors declare that the research was conducted in the absence of any commercial or financial relationships that could be construed as a potential conflict of interest.

## Publisher’s note

All claims expressed in this article are solely those of the authors and do not necessarily represent those of their affiliated organizations, or those of the publisher, the editors and the reviewers. Any product that may be evaluated in this article, or claim that may be made by its manufacturer, is not guaranteed or endorsed by the publisher.

## Abbreviations

ccRCC, clear cell renal cell carcinoma; DEGs, differentially expressed genes; DFS, disease-free survival; DSS, disease-specific survival; FUSCC, Fudan University Shanghai Cancer Center; GEO, Gene Expression Omnibus; MTAP, methylthioadenosine phosphorylase; PRMT5, protein arginine methyltransferase; OS, overall survival; TIME, tumor immune microenvironment; TME, tumor microenvironment; PFS, progression-free survival; RCC, renal cell carcinoma; sRCC, renal cell carcinoma with sarcomatoid dedifferentiation; TCGA, The Cancer Genome Atlas.
