# Supplementary figures and images for: Genomic alteration of MTAP/CDKN2A predicts sarcomatoid differentiation and poor prognosis and modulates response to immune checkpoint blockade in renal cell carcinoma
Source: Front Immunol. 2022 Aug 1;13:953721. doi: 10.3389/fimmu.2022.953721 (PMC9376285; doi:10.3389/fimmu.2022.953721)

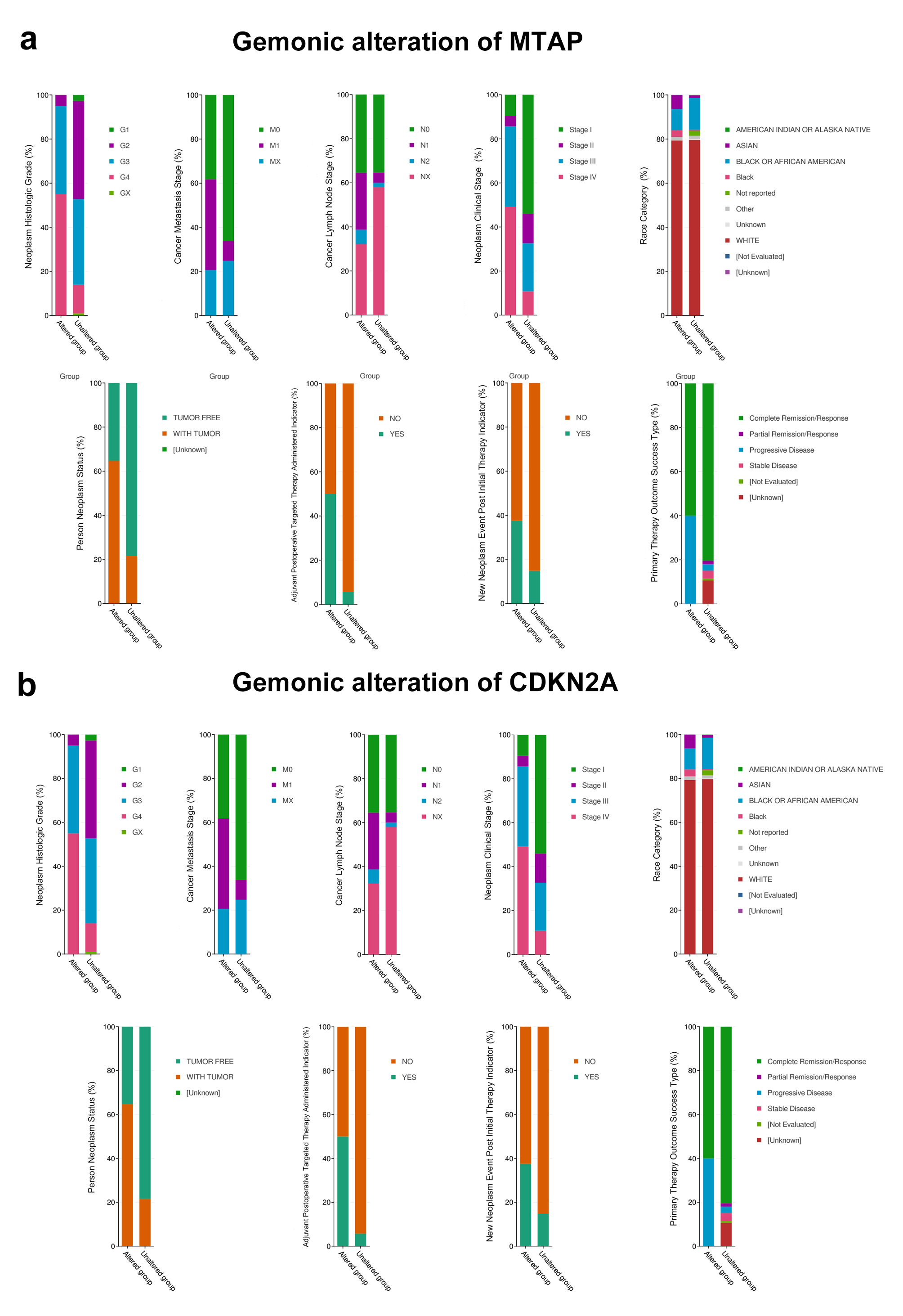

Supplement: Supplementary file 1 [file Image_1.tif]

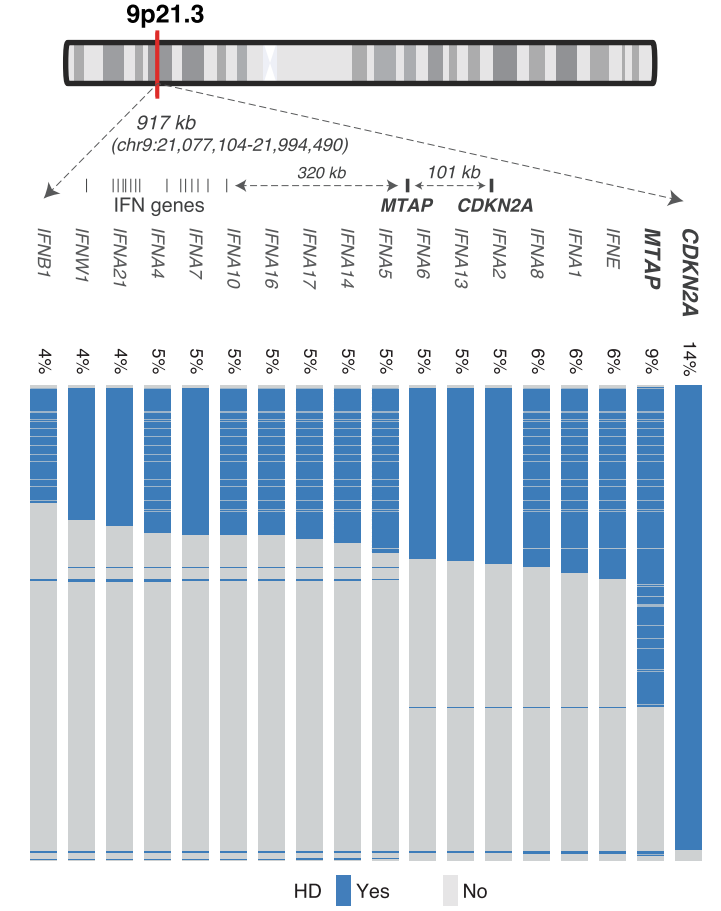

Supplement: Supplementary file 2 [file Image_2.png]
